# Association between overweight and growth hormone secretion in patients with non-functioning pituitary tumors

**DOI:** 10.1371/journal.pone.0267324

**Published:** 2022-04-22

**Authors:** Yasufumi Seki, Atsuhiro Ichihara

**Affiliations:** Department of Endocrinology and Hypertension, Tokyo Women’s Medical University, Tokyo, Japan; National Institute of Child Health and Human Development (NICHD), NIH, UNITED STATES

## Abstract

**Introduction:**

Growth hormone (GH) deficiency (GHD) is often complicated by non-functioning pituitary tumors (NFPTs); however, its prevalence remains unclear because preoperative screening for GHD with provocative tests is not recommended. Accordingly, we attempted to clarify the characteristics of GHD in unoperated patients with NFPT.

**Materials and methods:**

We retrospectively reviewed adult patients with non-functioning pituitary adenoma (NFPA) and Rathke’s cyst who underwent preoperative GH-releasing peptide-2 (GHRP-2) tests from January 2013 to December 2016. We investigated the association between peak GH response to GHRP-2 and background characteristics.

**Results:**

Among 104 patients (85 NFPA and 19 Rathke’s cysts), 45 (43%) presented severe GHD, as diagnosed using GHRP-2 tests. Body mass index (*β* = -0.210, *P* = 0.007), free thyroxine (*β* = 0.440, *P* < 0.001), and tumor height (*β* = -0.254, *P* < 0.001) were significant variables for determining the peak GH response to GHRP-2 in multiple regression analyses. Overweight (odds ratio, 3.86; 95% confidence interval, 1.02–14.66) was significantly associated with severe GHD after adjustment for age, sex, creatinine, free thyroxine, tumor height and clinical diagnosis. The regression slopes between tumor height and peak GH response to GHRP-2 significantly differed between overweight patients and non-overweight individuals, as determined by analysis of covariance (*P* = 0.040). In the 48 patients who underwent postoperative GHRP-2 tests, severe postoperative GHD was significantly more common in overweight patients than non-overweight individuals (100% *vs*. 48%, *P* < 0.001).

**Conclusion:**

We observed a negative synergistic effect between overweight and tumor size on GH secretion in patients with NFPTs, indicating that GH provocation tests for diagnosing underestimated GHD could be considered in overweight unoperated patients with large NFPTs.

## Introduction

Growth hormone (GH) deficiency (GHD) is the most common hormonal deficit complicated by pituitary tumors [1], and it is known to cause obesity, dyslipidemia, and premature atherosclerosis [2]. The major causes of adult-onset GHD are non-functioning pituitary adenomas (NFPAs) and Rathke’s cysts [3]. Patients with GHD reportedly present higher mortality and cardiovascular events compared to that of age- and sex-matched controls [4,5]. Furthermore, GH supplementation therapy has been associated with a decreased risk of cardiovascular events [5]. GH supplementation therapy also results in improved lipoprotein metabolism, body composition and bone mineral density [6]. Therefore, an appropriate diagnosis of GHD is crucial for improving cardiovascular outcomes in patients with pituitary tumors.

GH secretion is typically altered earlier than other anterior pituitary hormones in relation to the increasing mass effect of a pituitary tumor [7]. Random serum GH and insulin-like growth factor 1 (IGF-1) levels cannot be solely employed to diagnose GHD. GH provocative tests are required in patients with pituitary tumors, except for those with multiple pituitary hormone deficiencies (≥3 pituitary hormone deficiencies) and low serum IGF-1 levels (< -2.0 standard deviation [SD] score) [2]. As GHD screening prior to surgery is not recommended [8–10], only one previous study has reported the prevalence of GHD using a GH provocative test in patients with non-functioning pituitary tumors (NFPT) [11]. The prevalence and clinical characteristics of GH secretion in patients with NFPT remain unclear. Clarifying the clinical characteristics of GHD may be useful for identifying patients at high risk of GHD and for the diagnosis which leads to appropriate GH supplementation therapies.

Herein, we aimed to clarify the association between GH secretion and clinical characteristics, especially tumor size and other metabolic parameters, in patients with NFPTs. We retrospectively evaluated the association between GH secretion assessed by peak GH response to GH-releasing peptide-2 (GHRP-2) and background characteristics before pituitary surgery in patients with NFPTs.

## Materials and methods

### Study population

In this retrospective cross-sectional study, we reviewed all available charts of patients with NFPA and Rathke’s cyst who were admitted to the Department of Endocrinology at Tokyo Women’s Medical University Hospital between January 2013 and December 2016. Adult patients who underwent GHRP-2 tests before their first pituitary surgery were included in the present study. Patients with an estimated glomerular filtration rate (GFR) <30 mL/min/1.73 m^2^ were excluded. Background clinical characteristics at the time of the GHRP-2 testing, including age, sex, height, body weight, body mass index (BMI), comorbidities, smoking habit, renal function, pituitary function, and tumor size, were retrieved. Overweight was defined as BMI ≥ 25 kg/m^2^. Renal function was evaluated according to the 2012 Kidney Disease Improving Global Outcomes guidelines [12]. This study was performed in accordance with the 1964 Declaration of Helsinki and its amendments. As the study was defined as one without human samples under the Japanese guidelines presented by the Ministry of Health, Labour and Welfare, written informed consent was not required, and we used our official institutional website as an opt-out method. This study was approved by the Ethics Committee of Tokyo Women’s Medical University Hospital (4856-R).

### GH-releasing peptide-2 test

All patients underwent GHRP-2 tests to determine GH secretion. In brief, pralmorelin hydrochloride (0.1 mg) (Kaken Pharmaceutical Co. Ltd., Tokyo, Japan) was intravenously injected at 9 a.m., and blood samples were collected before and 15, 30, and 45 min after the injection. Severe GHD was defined as a peak GH concentration of less than 9 ng/mL [13]. A GH cut-off value of 9 ng/mL with GHRP-2 corresponded to a GH value of 1.8 ng/mL with an ITT when the GH value was calibrated with the recombinant WHO 98/574 standard [13]. Although the GHRP-2 test was not included in the Endocrine Society Guidelines [14], the GHRP-2 test is considered safe [15] and is widely employed to diagnose severe GHD in Japan owing to its high sensitivity and specificity when compared to ITT [16].

### Assays and measurements

Serum creatinine levels, HbA1c levels as per the National Glycohemoglobin Standardization Program, and the urinary albumin to creatinine ratio were measured using standard laboratory methods at our clinical laboratory center. Estimated GFR was calculated using the formula developed by the Japanese Society of Nephrology [17]. Patient serum GH concentrations were measured using a specific enzyme immunoassay (Tosoh Bioscience, Tokyo, Japan), calibrated with the recombinant WHO 98/574 standard. In addition, patient serum IGF-1 concentrations were measured using an immunoradiometric assay, “Daiichi” (Fujirebio, Tokyo, Japan). The IGF-1 SD score was calculated based on age- and sex-specific normative IGF-1 data in the Japanese population [18]. Free thyroxine concentrations were measured using an electrochemiluminescence immunoassay “ECLusys FT4” (Roche Diagnostics GmbH, Mannheim, Germany).

### Statistical analyses

Statistical analyses were performed using JMP Pro 14.0 software (SAS Institute, USA). Data are represented as mean ± SD or median (interquartile range) for descriptive analyses. Data normality was analyzed by visual inspection of Q–Q plots and histograms. For parameters with normal distribution, statistical significance between groups was calculated using the unpaired Student’s t-test. For parameters with skewed distribution, significance was assessed using the Mann-Whitney U test. Moreover, the significance of categorical data was assessed using the χ^2^ test. Given the skewed distribution, the peak GH response to GHRP-2 was log-transformed for regression analyses. Univariate regression analyses were performed using the Pearson correlation coefficient, except for HbA1c, urinary albumin to creatinine ratio, and change in peak GH response to GHRP-2 before and after surgery, which were assessed using the Spearman correlation coefficient. In multiple regression analyses, explanatory factors were selected from the results of the univariate regression analyses. An analysis of covariance (ANCOVA) was used to determine the correlation between tumor height and peak GH response to GHRP-2 adjusted for overweight. Multivariable logistic regression models were used to calculate adjusted odds ratios with 95% CIs for severe GHD. The model was adjusted for age, sex, creatinine level, free thyroxine level, tumor size, and clinical diagnosis. A *P* value < 0.05 was established as significant.

## Results

### Characteristics of study participants

We identified 104 patients with NFPA and Rathke’s cysts who underwent a GHRP-2 test before their first pituitary surgery. The diagnoses were pathologically confirmed in 71 (68%) patients using the GHRP-2 test. Among 104 patients, 17 (16%) presented central adrenal insufficiency, 17 (16%) had central hypothyroidism, and 28 (27%) had hypogonadotropic hypogonadism. Overall, 10 (10%) patients received hydrocortisone, 7 (7%) received levothyroxine, and 95 (91%) demonstrated normal thyroid function. No patient received GH supplementation before the GHRP-2 test. Table 1 presents the characteristics of study participants. Levels of serum creatinine (*P* = 0.025) and hemoglobin A1c (*P* = 0.030) and numbers of male sex (*P* = 0.005), hypertension (*P* < 0.001), diabetes mellitus (*P* = 0.024) and severe GHD (*P* = 0.004) were significantly higher in the overweight subgroup than in the non-overweight subgroup. Peak GH after GHRP-2 injection (*P* = 0.001) was significantly lower in the overweight subgroup than in the non-overweight subgroup.

**Table 1:** Background characteristics.

	All N = 104	Overweight N = 29	Non-overweight N = 75	*P* (Overweight vs Non-overweight)
Age	58 ± 14	60 ± 14	58 ± 14	0.40
Sex				0.005
Male sex, N (%)	49 (47)	20 (69)	29 (39)	
Female sex, N (%)	55 (53)	9 (31)	46 (61)	
Clinical diagnosis				0.174
Non-functioning adenoma	85 (82)	26 (90)	59 (79)	
Rathke’s cyst	19 (18)	3 (10)	16 (21)	
Pathological diagnosis, N (%)				0.138
Gonadotroph adenoma	33 (32)	7 (24)	26 (35)	
Null cell adenoma	22 (21)	11 (38)	11 (15)	
Rathke’s cyst	11 (11)	3 (10)	8 (11)	
Others	5 (5)	1 (3)	4 (5)	
Not pathologically diagnosed	33 (32)	7 (24)	26 (35)	
Height, cm	161.7 ± 9.0	163.4 ± 9.7	161.0 ± 8.7	0.21
Body weight, kg	62.2 ± 12.3	73.9 ± 11.3	57.6 ± 9.3	< 0.001
BMI, kg/m^2^	23.6 ± 3.2	27.5 ± 2.2	22.1 ± 2.1	< 0.001
BMI categories				< 0.001
BMI < 25 kg/m^2^, N (%)	75 (72)	0 (0)	75 (0)	
25 kg/m^2^ ≤ BMI < 30 kg/m^2^, N (%)	26 (25)	26 (90)	0 (0)	
BMI ≥ 30 kg/m^2^, N (%)	3 (3)	3 (10)	0 (0)	
Hypertension, N (%)	44 (42)	21 (72)	23 (31)	< 0.001
Diabetes mellitus, N (%)	15 (14)	8 (28)	7 (9)	0.024
History of smoking, N (%)	47 (45)	17 (59)	30 (40)	0.087
Serum creatinine, mg/dL	0.75 ± 0.16	0.80 ± 0.15	0.73 ± 0.16	0.025
Estimated GFR, mL/min/1.73 m^2^	74.5 ± 14.3	71.7 ± 10.8	75.6 ± 15.4	0.22
CKD categories				0.101
30 mL/min/1.73 m^2^ < estimated GFR ≤ 60 mL/min/1.73 m^2^, N (%)	17 (16)	4 (14)	13 (17)	
60 mL/min/1.73 m^2^ ≤ estimated GFR < 90 mL/min/1.73 m^2^, N (%)	72 (69)	24 (83)	48 (64)	
Estimated GFR ≥ 90 mL/min/1.73 m^2^, N (%)	15 (14)	1 (3)	14 (19)	
Hemoglobin A1c, %	5.7 (5.5–6.1)	5.8 (5.6–6.4)	5.7 (5.4–6.0)	0.030
Peak GH after GHRP-2 injection, ng/mL	13.63 (3.22–26.82)	3.40 (1.30–15.92)	15.39 (4.64–34.64)	0.001
Severe GH deficiency (peak GH < 9 ng/mL), N (%)	45 (43)	19 (66)	26 (35)	0.004
IGF-1, ng/mL	96 (74–127)	90 (59–118)	102(80–128)	0.172
IGF-1 SD score	-1.1 ± 1.3	-1.2 ± 1.5	-1.0 ± 1.3	0.41
Free thyroxine, ng/dL	1.11 ± 0.24	1.04 ± 0.25	1.14 ± 0.23	0.064
Urinary albumin to creatinine ratio, mg/gCr	4.8 (3.1–10.7)	4.9 (3.6–18.6)	4.5 (2.8–8.9)	0.29
Tumor size				
Width, mm	20.8 ± 8.3	20.3 ± 7.7	20.9 ± 8.5	0.72
Height, mm	20.0 ± 9.3	19.9 ± 9.0	20.1 ± 9.5	0.94
Depth, mm	16.9 ± 7.2	17.1 ± 7.1	16.8 ± 7.3	0.83

Data are presented as mean ± SD or median (interquartile range [IQR]). BMI, body mass index; GFR, glomerular filtration rate; CKD, chronic kidney disease; GH, growth hormone; GHRP-2, growth hormone-releasing peptide-2; SD, standard deviation.

### Severe GHD and background characteristics

Severe GHD, defined as a peak GH response to GHRP-2 < 9 ng/mL, was noted in 45 of 104 (43%) patients. Moreover, the percentage of male patients with severe GHD was higher than those without severe GHD (64% *vs*. 34%, *P* = 0.003). Age did not significantly differ between patients with and without severe GHD (*P* = 0.36). In addition, hypertension, diabetes mellitus, and a history of smoking did not differ significantly between patients with and without severe GHD.

**Fig 1:**
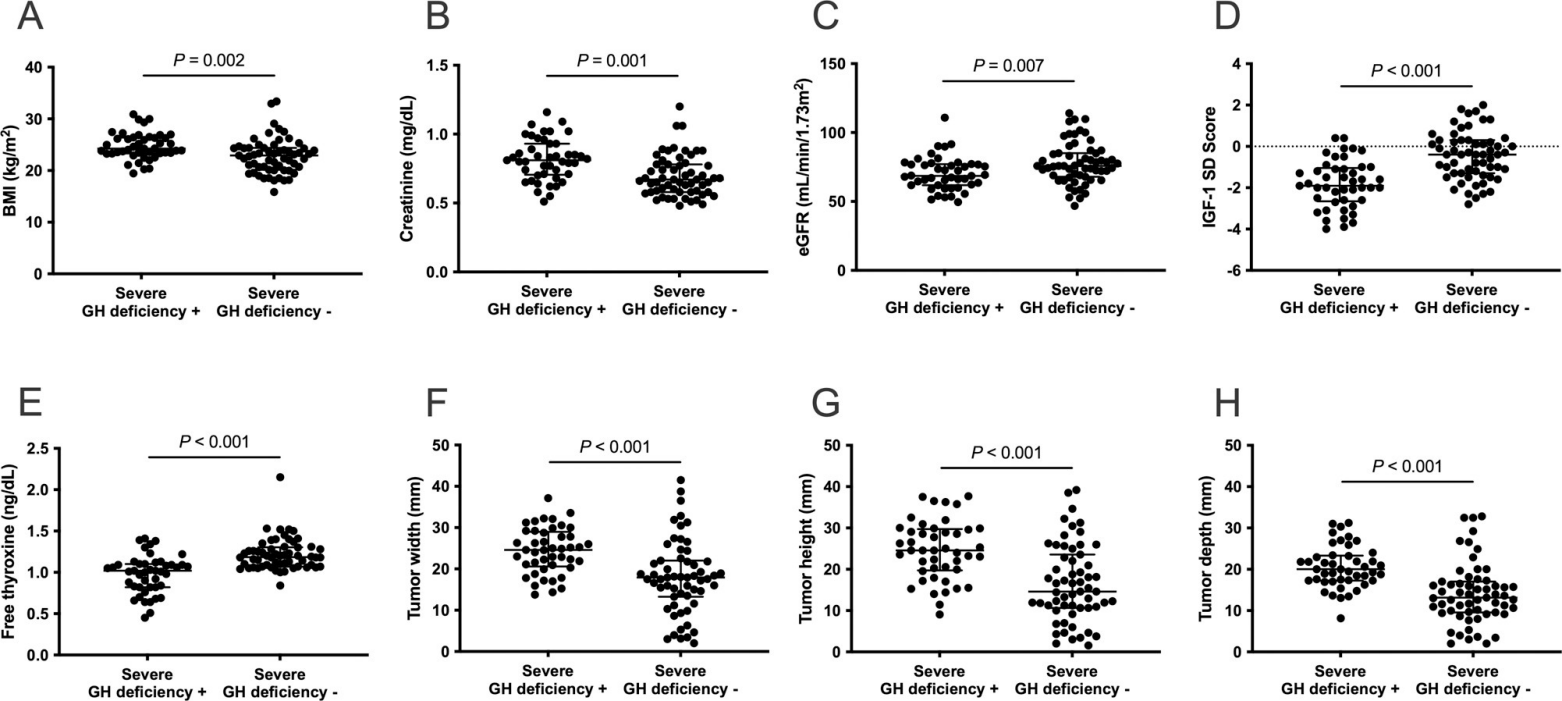
Background characteristics and severe GH deficiency (GHD). (A) BMI, (B) serum creatinine level, (F) tumor width, (G) height, and (H) depth are significantly higher in patients with severe GHD than in those without severe GHD. (C) Estimated GFR, (D) IGF-1 SD score, and (E) free thyroxine levels were significantly lower in patients with severe GHD than in those without severe GHD. GH, growth hormone; BMI, body mass index; GFR, glomerular filtration rate.

BMI was significantly higher in patients with severe GHD than in those without GHD (24.7 ± 2.6 *vs*. 22.8 ± 3.4 kg/m^2^, *P* = 0.002) (Fig 1A). Severe GHD was significantly more common in overweight patients than in non-overweight patients (66% *vs*. 35%, *P* = 0.008). Serum creatinine level was significantly higher (0.82 ± 0.15 *vs*. 0.70 ± 0.15 mg/dL, *P* < 0.001) (Fig 1B) and estimated GFR was significantly lower (70.2 ± 12.1 *vs*. 77.8 ± 15.1 mL/min/1.73 m^2^, *P* = 0.006) (Fig 1C) in patients with severe GHD than in those without severe GHD. Additionally, the IGF-1 SD score (-1.8 ± 1.2 *vs*. -0.5 ± 1.2, *P* < 0.001) (Fig 1D) and free thyroxine levels (0.98 ± 0.23 *vs*. 1.22 ± 0.19 ng/dL, *P* < 0.001) (Fig 1E) were significantly lower in patients with severe GHD than those without severe GHD. Tumor width (24.4 ± 5.5 *vs*. 18.0 ± 8.9 mm, *P* < 0.001) (Fig 1F), height (24.6 ± 7.1 *vs*. 16.6 ± 9.3 mm, *P* < 0.001) (Fig 1G), and depth (20.5 ± 5.1 *vs*. 14.1 ± 7.4 mm, *P* < 0.001) (Fig 1H) were significantly larger in patients with severe GHD than in those without severe GHD.

### Regression analyses with peak GH response to GHRP-2

**Fig 2:**
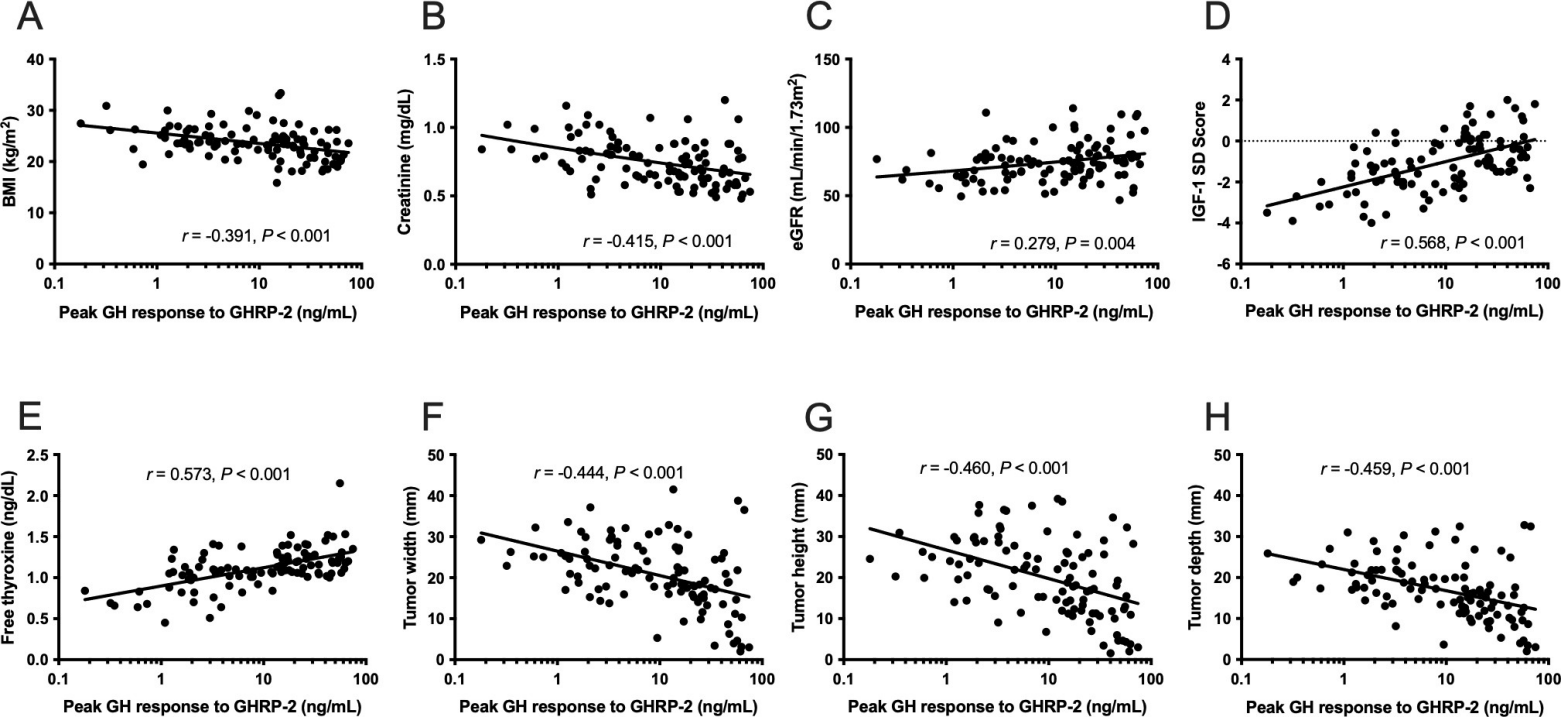
Background characteristics and peak GH response to GHRP-2. (A) BMI, (B) serum creatinine level, and (F) tumor width, (G) height, and (H) depth significantly correlate negatively with peak GH response to GHRP-2. (E) Estimated GFR, (D) IGF-1 SD score, and (E) free thyroxine levels significantly correlate positively with peak GH response to GHRP-2. GH, growth hormone; GHRP-2, growth hormone-releasing peptide-2; BMI, body mass index; GFR, glomerular filtration rate; IGF-1 SD, insulin-like growth factor 1; SD, standard deviation.

In univariate regression analyses, the peak GH response to GHRP-2 significantly and negatively correlated with BMI, serum creatinine level, and tumor diameter, and it was positively correlated with estimated GFR, IGF-1 SD score, and free thyroxine levels (Fig 2). The peak GH response to GHRP-2 did not significantly correlate with age (*r* = -0.074, *P* = 0.46), hemoglobin A1c (HbA1c) (*r*_*s*_ = -0.086, *P* = 0.40), or urinary albumin to creatinine ratio *(r*_*s*_ = 0.124, *P* = 0.29).

In the multiple regression analyses, BMI (*β* = -0.210, *P* = 0.007), free thyroxine (*β* = 0.440, *P* < 0.001), and tumor height (*β* = -0.254, *P* < 0.001) were significant variables for determining the logarithm of the peak GH response to GHRP-2 (Table 2).

**Table 2:** Multiple regression analyses with peak GH response to GHRP-2.

	β	*P*	VIF
Age	0.005	0.94	1.10
Sex (male)	-0.047	0.68	2.68
BMI	-0.210	0.007	1.24
Creatinine	-0.193	0.095	2.77
Free thyroxine	0.440	< 0.001	1.13
Tumor height	-0.254	< 0.001	1.18

GH, growth hormone; GHRP-2, growth hormone-releasing peptide-2; BMI, body mass index; VIF, Variance inflation factor.

### Effect of overweight on peak GH response to GHRP-2

Multivariable logistic analyses were performed to investigate the effect of overweight on severe GHD (Table 3). Overweight (BMI ≥ 25 kg/m^2^) was significantly associated with severe GHD when the model was adjusted for free thyroxine and tumor height (odds ratio, 4.67; 95% confidence interval [CI], 1.43–15.26). Furthermore, on adjusting the model for age, sex, creatinine, and clinical diagnosis, overweight was significantly associated with severe GHD (odds ratio, 3.86; 95% CI, 1.02–14.66).

**Table 3:** Association between overweight and severe GH deficiency.

	Crude		Adjusted for free thyroxine and tumor height		Adjusted for age, sex, creatinine, free thyroxine, tumor height and clinical diagnosis	
	Odds ratio (95% CI)	*P*	Odds ratio (95% CI)	*P*	Odds ratio (95% CI)	*P*
Overweight	3.58 (1.45–8.82)	0.006	4.67 (1.43–15.26)	0.012	3.86 (1.02–14.66)	0.047

CI, confidence interval; GH, growth hormone; BMI, body mass index.

**Fig 3:**
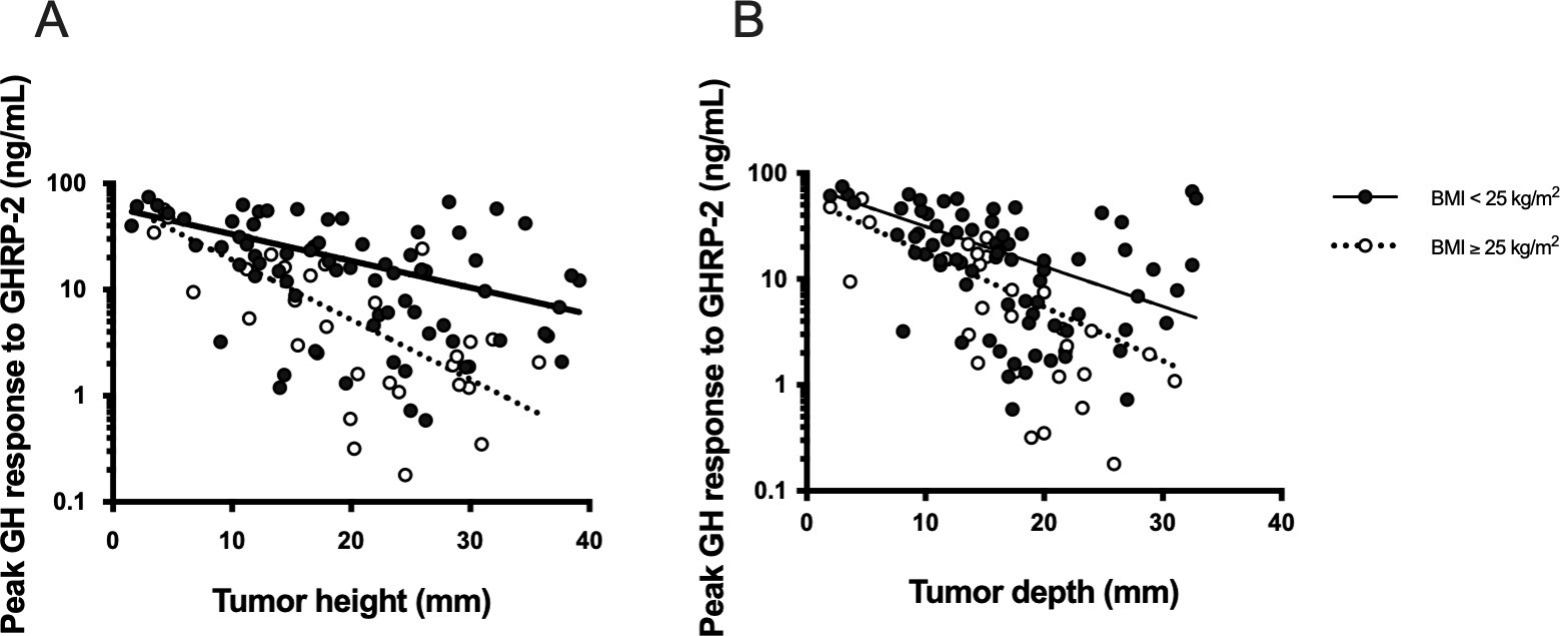
Peak GH response to GHRP-2 and tumor size in overweight and non-overweight patients. (A, B) Open circles and closed circles represent peak GH response to GHRP-2 in overweight (BMI ≥ 25 kg/m^2^) and non-overweight patients (BMI < 25 kg/m^2^), respectively. The dotted and solid lines represent linear regression curves between peak GH response to GHRP-2 and tumor height (A) and tumor depth (B) in overweight (BMI ≥ 25 kg/m^2^) and non-overweight patients (BMI < 25 kg/m^2^), respectively. GH, growth hormone; GHRP-2, growth hormone-releasing peptide-2; BMI, body mass index.

We evaluated the effect of overweight on the correlation between tumor size and GH secretion. Fig 3 presents a negative correlation between tumor height (Fig 3A) and tumor depth (Fig 3B) and peak GH response to GHRP-2. We performed ANCOVA to evaluate the effects of tumor size and overweight on the peak GH response to GHRP-2 and detected significant interaction effects between overweight and tumor height (*β* = -0.191, *P* = 0.040) in Model 1and between overweight and tumor depth (β = -0.244, *P* = 0.007) in Model 2 (Table 4). Similarly, not only BMI but also significant interaction effects between BMI and tumor height (β = -0.209, *P* = 0.014) in Model 3 and tumor depth (β = -0.259, *P* = 0.002) in Model 4 were observed in multiple regression analyses with peak GH response to GHRP-2 (Table 4).

**Table 4:** Multiple regression analyses with peak GH response to GHRP-2.

	β	*P*
Model 1		
Interaction effect (overweight × tumor height)	-0.191	0.040
Overweight	-0.359	< 0.001
Tumor height	-0.557	< 0.001
Model 2		
Interaction effect (overweight × tumor depth)	-0.244	0.007
Overweight	-0.340	< 0.001
Tumor depth	-0.566	< 0.001
Model 3		
Interaction effect (BMI × tumor height)	-0.209	0.014
BMI	-0.449	< 0.001
Tumor height	-0.448	< 0.001
Model 4		
Interaction effect (BMI × tumor depth)	-0.259	0.002
BMI	-0.408	< 0.001
Tumor depth	-0.478	< 0.001

BMI, body mass index; GH, growth hormone; GHRP-2, growth hormone-releasing peptide-2.

### Peak GH response to GHRP-2 after pituitary surgery

In 48 patients (42 NFPA and 6 Rathke’s cyst), postoperative GHRP-2 tests were performed one year after their first pituitary surgeries. No patient received GH supplementation therapy. Severe postoperative GHD was significantly more frequent in patients with severe preoperative GHD than in those without severe preoperative GHD (83% *vs*. 33%, *P* = 0.001) (Table 5). Body weight (*P* = 0.085) and BMI (*P* = 0.083) were not significantly altered before and after surgery (Table 6). All patients who were overweight before surgery (N = 15) showed severe postoperative GHD, and the incidence rate was significantly higher in overweight patients than in non-overweight patients (100% *vs*. 48%, *P* < 0.001) (Table 5). Preoperative BMI significantly correlated with the postoperative peak GH response to GHRP-2 (*r* = -0.355, *P* = 0.013), but did not correlate with the change in peak GH response to GHRP-2 before and after the surgery (*r*_*s*_ = 0.045, *P* = 0.76). On the other hand, 47 patients presented large tumors with tumor heights ˃1 cm. Tumor height was not significantly associated with postoperative peak GH response to GHRP-2 (*r* = -0.241, *P* = -0.099) or the change in peak GH response to GHRP-2 before and after the surgery (*r*_*s*_ = -0.180, *P* = 0.22).

**Table 5:** Association between preoperative GHD and overweight and postoperative GHD.

	Postoperative GH status		*P*
	Severe GHD+(N = 31)	Severe GHD-(N = 17)	
Preoperative GH status			0.001
Severe GHD+ (N = 30)	25 (83%)	5 (17%)	
Severe GHD- (N = 18)	6 (33%)	12 (67%)	
Preoperative overweight			< 0.001
Overweight (N = 15)	15 (100%)	0 (0%)	
Non-overweight (N = 33)	16 (48%)	17 (52%)	

GH, growth hormone; GHD, growth hormone deficiency.

**Table 6:** Body weight and BMI before and after pituitary surgery.

	Before surgery	After surgery	*P*
Body weight, kg	61.2 ± 11.2	62.0 ± 12.0	0.085
BMI, kg/m^2^	23.8 ± 2.6	24.0 ± 2.7	0.083

Data are presented as mean ± SD. BMI, body mass index.

## Discussion

In this retrospective study, severe GHD was observed in 43% of patients with NFPA and Rathke’s cysts in accordance with the GHRP-2 test results. The peak GH response to GHRP-2 significantly and negatively correlated with BMI, serum creatinine levels, and tumor size while positively correlating with eGFR, serum IGF-1 level, and IGF-SD score. Multiple regression analyses revealed that BMI, free thyroxine, and tumor height were significant variables in determining the logarithm of the peak GH response to GHRP-2. In the logistic regression analyses, overweight (BMI ≥ 25 kg/m^2^) was significantly associated with severe GHD after adjustment for potential predictors. Furthermore, based on ANCOVA results for peak GH response to GHRP-2, the regression slope between tumor height and GH secretion differed significantly between overweight and non-overweight patients. In patients who underwent postoperative GHRP-2 tests, all overweight patients presented severe GHD one year after pituitary surgery.

Herein, we observed two important characteristics of GHD in patients with NFPTs. First, the incidence of overweight might be an important clinical characteristic for estimating GHD in patients with NFPT. As obesity is reportedly associated with lower GH responses in GH provocative tests [19–22], an alternate GH cut-off for diagnosing GHD was recommended for assessing patients with a BMI ˃ 30 kg/m^2^, as well as in patients with a BMI of 25–30 kg/m^2^ and a low pretest probability [2]. In the present study, most patients had a BMI < 30 kg/m^2^. In addition, regression slopes between tumor height and peak GH response to GHRP-2 significantly differed between overweight and non-overweight patients. This indicates that the effect of tumor size on GH secretion differed between overweight and non-overweight patients. However, this negative synergistic effect of overweight and tumor size on GH secretion could not be explained solely by an obesity-related low GH response. Even if the BMI-related cut-offs, such as those for insulin tolerance tests [23], were used, the two regression slopes between tumor size and GH secretion were not changed because those cut-offs were not to describe the different slopes. In overweight patients, GH secretion might be altered due to pituitary compression caused by NFPT. Hyperinsulinemia, which might be associated with low GH secretion during obesity [24], may accelerate altered GH secretion by pituitary compression.

Second, GHD is common in patients with NFPT. In the present study, 45 of 104 (43%) patients with NFPT had a low GH response to GHRP-2, which is considered equivalent to severe GHD. As small pituitary tumors were not associated with hormone deficiencies [25], few studies have shown the prevalence of GHD in preoperative patients with NFPTs, which was confirmed by provocative tests. Our results were similar to those of a study that detected GHD, as confirmed by the GHRH-arginine test, in 19 (50%) of 38 patients with NFPT and normal IGF-1 levels [11]. In addition, severe GHD, as confirmed by the GHRP-2 test, was reported in 36 (47%) of 76 patients with NFPA [26].

These findings suggest that GH provocative tests could be considered in overweight patients with large NFPTs who would not undergo surgeries. In NFPT, indications for pituitary surgery depend on closeness to optic chiasm and endocrine deficits [10]. However, endocrine assessments typically do not include the GH axis [8], although postoperative GHD has been focused [27]. Therefore, most unoperated patients with NFPTs have not received appropriate assessment for GH secretion and GHD in those patients may be underestimated. Based on our findings, GH provocation tests for diagnosing GHD could be considered in overweight unoperated patients with large NFPTs.

This study has some limitations. First, we could not establish a causal relationship between the low GH response to GHRP-2 and overweight due to the retrospective and observational nature of the present study. Compression of the pituitary by NFPT can increase BMI through GHD, and obesity itself can also result in a low GH response to GHRP-2. Second, the diagnosis of GHD was confirmed using the GHRP-2 test. A high correlation between the GHRP-2 test and the insulin tolerance test (ITT) (*r* = 0.8745, *P* < 0.0001) has been reported in adult preoperative Japanese patients with pituitary tumors [16], and although the GHRP-2 test has been approved in Japan, it is not recommended in the guidelines of the Endocrine Society [14]. We did not compare the peak GH response to GHRP-2 and other GH secretagogues in this study. Third, the thyroid function was abnormal in 9% of patients in the present study. Reportedly, hypothyroidism can be associated with a lower peak GH response to GH secretagogues [28]. Although we performed multivariate analyses adjusted for thyroid function, the thyroid function might impact the peak GH response to GHRP-2.

In conclusion, this retrospective study revealed the synergistic effect of overweight and tumor size on the low peak GH response to GHRP-2 in patients with NFPTs. Because GHD in unoperated patients may be underestimated, GH provocation tests for diagnosing GHD could be considered in overweight unoperated patients with large NFPTs.

## Supporting information

S1 Dataset Dataset of the study. (XLSX)
